# Condom use and prevalence of syphilis and HIV among female sex workers in Andhra Pradesh, India – following a large-scale HIV prevention intervention

**DOI:** 10.1186/1471-2458-11-S6-S1

**Published:** 2011-12-29

**Authors:** Hari Kumar Rachakulla, Venkaiah Kodavalla, Hemalatha Rajkumar, SPV Prasad, Srinivasan Kallam, Prabuddhagopal Goswami, Jayesh Dale, Rajatashuvra Adhikary, Ramesh Paranjape, GNV Brahmam

**Affiliations:** 1 Division of Community Studies, National Institute of Nutrition (NIN), Hyderabad, India; 2 Department of Bio-statistics, National Institute of Nutrition (NIN), Hyderabad, India; 3 Department of Micro-Biology, National Institute of Nutrition (NIN), Hyderabad, India; 4 FHI, New Delhi, India; 5 National AIDS Research Institute (NARI), Pune, India

## Abstract

**Background:**

Avahan, the India AIDS initiative began HIV prevention interventions in 2003 in Andhra Pradesh (AP) among high-risk groups including female sex workers (FSWs), to help contain the HIV epidemic. This manuscript describes an assessment of this intervention using the published Avahan evaluation framework and assesses the coverage, outcomes and changes in STI and HIV prevalence among FSWs.

**Methodology:**

Multiple data sources were utilized including Avahan routine program monitoring data, two rounds of cross-sectional survey data (in 2006 and 2009) and STI clinical quality monitoring assessments. Bi-variate and multivariate analyses, Wald Chi-square tests and multivariate logistic regressions were used to measure changes in behavioural and biological outcomes over time and their association.

**Results:**

Avahan scaled up in conjunction with the Government program to operate in all districts in AP by March 2009. By March 2009, 80% of the FSWs were being contacted monthly and 21% were coming to STI services monthly. Survey data confirmed an increase in peer educator contacts with the mean number increasing from 2.9 in 2006 to 5.3 in 2009. By 2008 free and Avahan-supported socially marketed condoms were adequate to cover the estimated number of commercial sex acts, at 45 condoms/FSW/month. Consistent condom use was reported to increase with regular (63.6% to 83.4%; AOR=2.98; p<0.001) and occasional clients (70.8% to 83.7%; AOR=2.20; p<0.001). The prevalence of lifetime syphilis decreased (10.8% to 6.1%; AOR=0.39; p<0.001) and HIV prevalence decreased in all districts combined (17.7% to 13.2%; AOR 0.68; p<0.01). Prevalence of HIV among younger FSWs (aged 18 to 20 years) decreased (17.7% to 8.2%, p=0.008). A significant increase in condom use at last sex with occasional and regular clients and consistent condom use with occasional clients was observed among FSWs exposed to the Avahan program. There was no association between exposure and HIV or STIs, although numbers were small.

**Conclusions:**

The absence of control groups is a limitation of this study and does not allow attribution of changes in outcomes and declines in HIV and STI to the Avahan program. However, the large scale implementation, high coverage, intermediate outcomes and association of these outcomes to the Avahan program provide plausible evidence that the declines were likely associated with Avahan. Declining HIV prevalence among the general population in Andhra Pradesh points towards a combined impact of Avahan and government interventions.

## Introduction

India was estimated to have nearly 2.4 million persons living with HIV in 2009 [1-3]. It is now widely accepted that India, like many other Asian countries, has many concentrated HIV epidemics [4] with the major driving force being the size of the female sex worker (FSW) population and their clients [5,6]. The Indian National AIDS Control Organisation (NACO) has been implementing targeted interventions with high-risk groups (HRGs) such as female sex workers (FSWs), men who have sex with men (MSM), transgenders (TGs), injecting drug users (IDUs) as well as with bridge groups to contain the epidemic in India [7].

Avahan, the India AIDS Initiative, initiated a focused HIV prevention program in the historically six high prevalence states in India starting in 2003 [8]. This large-scale HIV prevention program targets FSWs, their clients, high-risk MSM (many of whom report selling sex) and TGs, IDUs and long-distance truck drivers along the national highways. The Avahan intervention districts in each state were selected in consultation with respective states governments to complement their HIV prevention efforts among HRGs and bridge groups to influence changes in the determinants of HIV infections and thereby prevent new infections in the general population [9]. The Avahan program’s main strategies were to achieve high coverage and deliver a package of proven prevention services, addressing proximate and distal determinants of HIV risk [10]. To achieve this, Avahan supported peer-led outreach and education, promotion and availability of condoms, management of sexually transmitted infections (STIs) and interventions to address structural and environmental barriers [11].

The state of Andhra Pradesh, the fifth most populous south-eastern state in India, is one of the focus Avahan states. Andhra Pradesh is one of the high HIV prevalence Indian states, with HIV prevalence estimated at 2% among women attending urban antenatal clinics and 20 of the 23 districts reporting HIV prevalence above 1% till 2005 [12]. The State AIDS Control Society in Andhra Pradesh was established during the phase II of the National AIDS Control Programme (1999-2007), with early interventions for FSWs and MSM in 2001 [12]. The Avahan program has been the major partner of the Andhra Pradesh Government AIDS Control Society and has been implementing the project among HRGs, as the only (solo), major or minor program partner in all 23 districts in the three regions of the state since October 2004. The Avahan coverage of the target population in the district varied and was decided upon in consultation with the state program. Table 1 provides the list of districts with the estimated number of FSWs and targeted coverage by Avahan program.

**Table 1:** District-level Avahan intended coverage and history of intervention in Andhra Pradesh

Districts	District population^1^ (thousands)	Estimated size of FSW in districts^2^	**Avahan intended coverage^3^**	History / type of intervention coverage*	IBBA sample	
					
					R-I	R-II
Adilabad	2,479	3,215	51%	Not first but equal		
					
Anantapur	3,639	12,611	100%	First and solo		
					
Chittoor	3,735	9,141	81%	Not first but major	401	398
					
Cudappah	2,573	2,183	45%	Not first but major		
					
East Godavari	4,872	8,795	19%	Not first and minor	422	401
					
Guntur	4,405	9,223	66%	Not first but major	405	405
					
Hyderabad	3,686	4,611	19%	Not first and minor	399	401
					
Karimnagar	3,477	3,880	100%	First and solo	412	402
					
Khammam	2,565	4,771	100%	First and solo		
					
Krishna	4,218	6,578	37%	Not first but major		
					
Kurnool	3,512	6,378	65%	Not first but major		
					
Mahboobnagar	3,506	1,090	28%	Not first and minor		
					
Medak	2,662	1,950	100%	First and solo		
					
Nalgonda	3,238	1,612	100%	First and solo		
					
Nellore	2,659	4,178	100%	Not first but solo		
					
Nizamabad	2,342	2,154	100%	First and solo		
					
Prakasam	3,054	8,424	44%	Not first but equal	404	408
					
Rangareddi	3,506	2,342	100%	First and solo		
					
Srikakulam	2,528	1,805	100%	First and solo		
					
Vishakhapatnam	3,789	4,140	55.3%	Not first but equal	411	409
					
Vizianagaram	2,245	2,706	67%	Not first but major		
					
Warangal	3,231	4,733	80%	Not first but major	417	401
					
West Godavari	3,796	5,871	63%	Not first but major		

**Source: 1-**Census 2001; **2-**District mapping data **3-**Avahan CMIS 2009

**First and solo: 1)** Prior to Avahan (i.e. 2003), there was no intervention for FSW; **2)** Currently Avahan is the only intervention in the district.

**Not first but solo:** Prior to Avahan (i.e. 2003), there were intervention for FSW; 2) Currently Avahan is the only intervention in the district

**Not first but major:** Prior to Avahan (i.e. 2003), FSW intervention existed in the districts and currently Avahan covers more than 50% of the mapped FSW population in the district

**Not first but equal:** Prior to Avahan (i.e. 2003), FSW intervention existed in the districts and currently Avahan covers 30 - 50% of the mapped FSW population in the district

**Not first and minor:** Prior to Avahan (i.e. 2003), FSW intervention existed in the districts; 2) Avahan covers less than 30 of the mapped FSW population in the district

Avahan’s evaluation framework was based on recent approaches for large-scale public health programs [13] and followed the program’s logic model [14] to assess scale-up and coverage, changes in intermediate outcomes, changes in general population HIV prevalence and association of these with HRGs and Avahan’s possible association with these changes [9]. To answer the questions posed in the evaluation framework, multiple rounds of cross-sectional surveys (termed Integrated Behavioural and Biological Assessments – IBBAs) were planned and conducted to assess the intermediate outcomes and separate modelling exercises to answer questions on impact.

The current paper presents an assessment of the Avahan program for FSWs in the state of Andhra Pradesh, using the above evaluation framework [9] and responds to its questions using program monitoring data and validation of these using independent survey data.

## Methodology

### Analytical framework for assessment

An analytical framework for the proposed assessment was developed drawing directly from the Avahan program evaluation design [9]. This framework is detailed in Table 2 and addresses the assessment questions step-by-step following the logical sequence of program implementation (process and output indicators), intermediate outcomes and contributions of Avahan. The specific aims were to: (a) examine the scale, intensity (based on availability and utilization of services) and quality of Avahan coverage; (b) assess the intermediate outcome of consistent condom use; (c) assess changes in prevalence of STIs including HIV; and (d) examine the association of Avahan exposure with changes in condom use and STI prevalence.

**Table 2:** Framework for assessment

Assessment question	Indicator	Data source
**1. Is coverage of Avahan adequate?**	**A. Scale**	
	a. ***Geographical coverage***	
	Description of roll out in number of districts and change in number of implementing NGOs over time	CMIS
	b. ***Proportion of FSWs ever contacted and ever visited clinic***	
	Number of FSWs ever contacted by Avahan peer educator and ever visited Avahan program clinics using the estimated size of FSWs as on March 2009 (data is from the Avahan CMIS and are based on mapping exercises taken up by Avahan NGOs once every 12 to 24 months in the coverage districts). This was compared with IBBA data among FSWs who reported ‘ever been contacted by Avahan program’ and ‘ever visited Avahan program clinics’	CMIS and IBBA
	c. ***Monthly contacts by peer educators and monthly visits for STI consultations***	
	FSWs contacted monthly by Avahan PEs and consultations for STIs by visit for STI in the month.	CMIS
	d. ***Proportion contacted in last month***	
	Percentage of FSWs from IBBA who reported that they had been contacted by Avahan peer educators in the month preceding survey using the estimated population size of FSWs as on March 2009	IBBA
	
	**B. Intensity**	
	***a. Number of peer educator/outreach worker and Ratio of sex worker to peer educator***	
	The total number of active outreach workers and peer educators in the Avahan intervention areas across implementation districts in Andhra Pradesh; and number of estimated FSWs covered per peer educators in the coverage area	CMIS and Condom Social Marketing data (CSM)
	***b. Condom distribution and availability***	
	**1.** Absolute number of free condom distributed by Avahan programme annually and condom sales from project supported condom social marketing by program between 2005 and 2008	CMIS, CMS and IBBA
	**2.** Condom need analysis: Ratio of average monthly condoms available per FSW- total condoms distributed by Avahan and available through project supported condom social marketing sales divided by the estimated number of FSWs in area covered by Avahan; and ratio of number of condoms distributed to monthly commercial sex acts per FSW where sex acts are calculated based on mean number of commercial clients per FSW per week reported in IBBA multiplied by total estimated number of FSWs covered by Avahan multiplied by four to get monthly sex acts.	IBBA
	**3.** Proportion of FSW reporting source of obtaining condom last time from outreach worker / peer educator / non-governmental organization	

	***c. Frequency of contact by peers***	
	FSW reporting number of times they were contacted by peer educators in the month preceding the survey	IBBA
	***d. Frequency of visit to clinic***	
	FSW reporting number of times visiting the Avahan program clinics for STI services	Individual level CMIS data
	**C. Quality**	
	***a. Improvement of quality of clinic services***	
	Quality monitoring of STI clinical services of Avahan clinics using a supervisory tool for all Andhra Pradesh intervention districts from 2005 to 2009.	Clinic data

**2. Has there been an increase in condom use in HRGs?**	**Change in condom use pattern**	
	a. Proportion of FSW reporting consistent condom use with occasional clients (clients who were unknown to the FSW) and regular clients (who visited repeatedly and were thus known to FSW) from two rounds of IBBAs	IBBA
	b. Proportion of FSW reporting no unprotected sex acts with clients from two rounds of IBBA	
	c. Proportion of FSW reporting consistent condom use with regular partners (husband or steady boyfriend of FSWs) from two rounds of IBBA	

**3. Has there been reduction in STIs and new HIV infections?**	**Change in STI prevalence and visits to clinic with STI symptoms**	
	a. STI prevalence (reactive syphilis serology, high-titre syphilis, gonorrhoea (NG), chlamydia (CT), any STI (GC or CT or high-titre syphilis)	IBBA
	
	**Change in HIV prevalence and new HIV infections**	
	a. HIV prevalence among FSWs aggregated from eight districts in Andhra Pradesh in two rounds of IBBAs	IBBA
	b. HIV prevalence among FSWs who are in sex work for duration of less than one year (as a proxy for incidence)	
	c. HIV prevalence among FSWs in the age group of 18-20 years	

**4. Is Avahan exposure associated with increase in condom use and declining STIs?**	**Association of program exposure with intermediate outcomes and STIs**	
	Exposure to Avahan program was based on self-reported responses to three Avahan program core services during last one year Viz; (a) ever or last year contacted by peer educators; (b) ever visited Avahan STI clinics; and (c) received condoms from peer educators. A composite variable for ‘having received any one service’ was used.	IBBA
	a. Association of Avahan program with consistent condom use with commercial and non-commercial partners using pooled data from two rounds of IBBA	
	b. Program exposure, as defined above, its association with having any STI (NG, CT or high-titre syphilis)	

### Data sources

The present analysis used the following sources of data.

#### a. Avahan routine program monitoring data

Avahan developed a computerized management information system (CMIS) of outreach services [15,16] and of clinical services [17] through the course of program implementation. In each district, NGO partners implementing the Avahan program gathered and reported monthly data on program inputs and infrastructure, outreach services and clinical service utilization. Data were aggregated and reported to the lead implementing partner at the state level and a subset of indicators was aggregated centrally using the CMIS. Program monitoring data were used to construct trends to assess coverage and uptake of program services between 2005 and March 2009 [15,16]. The scope of this analysis included program monitoring data from all 23 districts of Andhra Pradesh.

#### b. Integrated Behavioural and Biological Assessments

**Figure 1:**
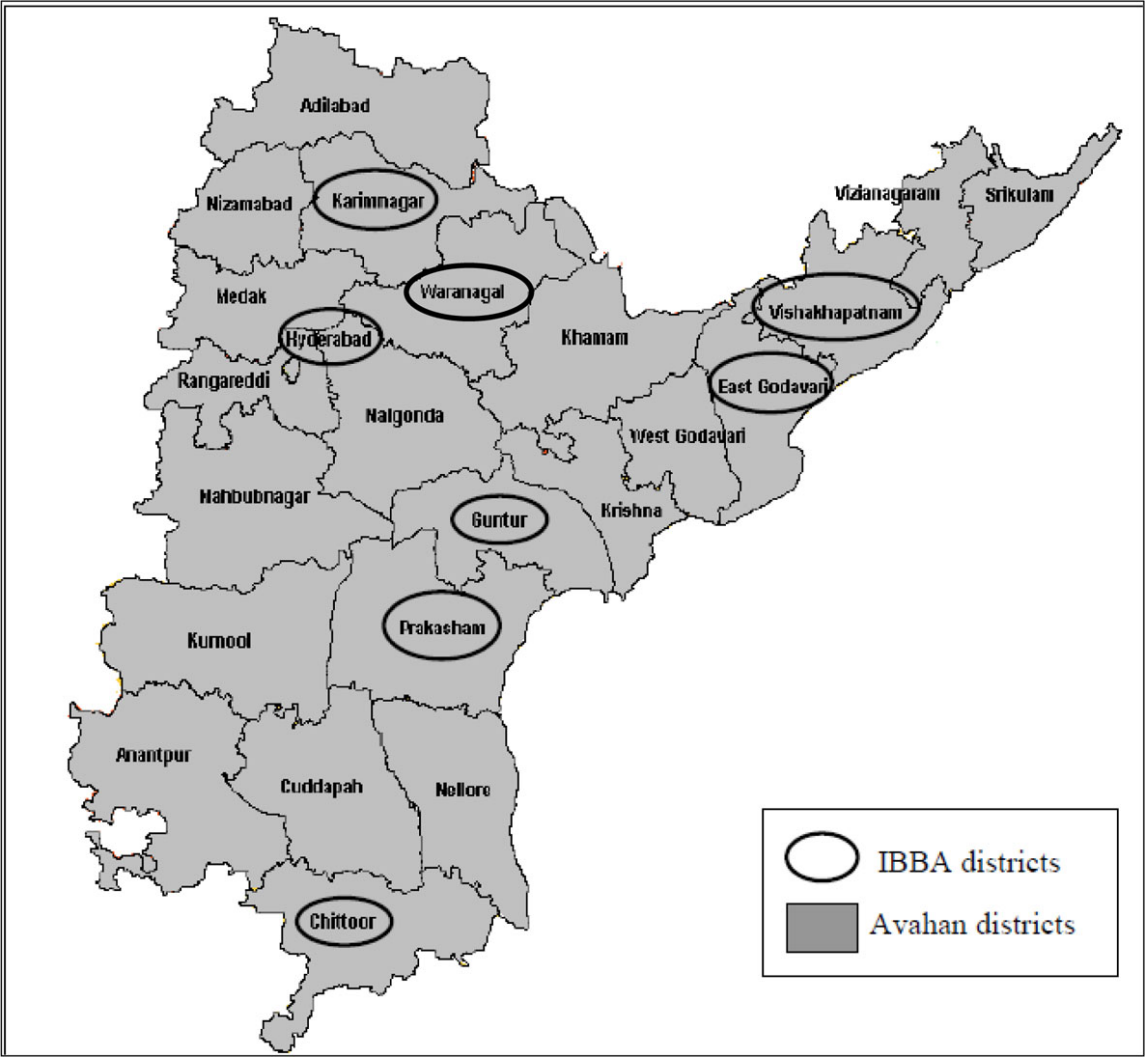
Map of Avahan Districts in Andhra Pradesh and IBBA Districts.

Two rounds of IBBAs were undertaken among FSWs in eight of the 23 Avahan intervention districts. Round 1 was conducted between November 2005 and December 2006 and Round 2 between March and October 2009. Districts were chosen purposively based on size of the FSW population and socio-cultural regions and included Chittoor, East Godavari, Guntur, Hyderabad, Karimnagar, Prakasam, Visakhapatnam and Warangal (Figure 1, Table 2). Both rounds used identical study methodologies. Probability-based sampling methods, such as conventional cluster sampling and time-location sampling, were used following a comprehensive sampling frame development exercise spanning the entire district [18,19]. Both rounds of IBBAs collected behavioural information and biological specimens to test for STIs including HIV. Fieldwork was conducted by research agencies under the guidance and supervision of the implementing State Indian Council of Medical Research (ICMR) Institute in Andhra Pradesh, the Indian National Institute of Nutrition (NIN) and National AIDS Research Institute (NARI). The international agency, FHI provided technical assistance to conduct both rounds of IBBA. The survey team was provided training on the survey protocol, questionnaire administration, sample collection and transport of biological samples. Appropriate ethical clearances were obtained prior to surveys. Complete details of the IBBA methodology are available in the paper by Saidel et al [18].

#### c. STI clinical quality monitoring assessments

A major component of Avahan was to ensure high quality and standardized STI services across implementation partners. A central STI capacity building team, led by FHI, developed a clinical quality monitoring tool to be completed in a participatory manner in Avahan clinics at periodic intervals. This tool comprising 80 questions using a five-point scale covering five performance areas of coverage, quality of clinic and services, referral networks, community involvement and technical support. The quality monitoring assessments of STI clinical services were conducted quarterly by an external team in about 10% to 20% of program-supported clinics. A detailed methodology for these assessments has been described elsewhere [20]. Total scores were calculated and a correlation matrix was used to examine significant change in quality scores between years using STATA 11® (Stata Corporation, College Station, TX).

### Operational definitions and assumptions

#### 1. Is coverage of Avahan adequate?

Coverage was defined on the basis of availability, utilization and coverage of HIV prevention services for FSWs [15]. The adequacy of coverage was defined based on the Avahan target for saturated coverage, set at services being provided to 80% of the estimated number of FSWs in the intervention districts. Estimates of FSW denominators used from the Avahan CMIS were based on mapping exercises undertaken by Avahan NGOs once every 12 to 24 months [15,16].

Routine monitoring data from the Avahan CMIS were used to assess availability and utilization of services (Table 2). The Avahan target for outreach contacts was a minimum of one contact per month; whereas the target for clinic visits was once per quarter (about 33% of the denominator per month) for STI consultations. As an independent assessment of coverage (evaluated coverage) and to validate the utility of the CMIS data, coverage information was elicited in the IBBA.

Intensity was defined as the frequency of exposure to intervention. Manpower to achieve intensity program was measured using two Avahan CMIS indicators: (a) change in staff numbers, such as numbers of outreach workers and peer educators who made outreach contacts with FSWs; and (b) the ratio of peer educators to the estimated number of FSWs (the program target ratio is 1:50) [14].

Intensity of exposure was assessed also based on total number of condoms distributed and available to cover commercial sex acts, frequency of outreach contacts and frequency of STI clinic service utilization. Avahan services included promotion and provision of free and socially marketed condoms based on the estimated need. Data on free condoms distributed to FSWs by peer educators, outreach workers, program clinics and condom depots were tracked yearly from the Avahan CMIS and data on annual condom sales from program supported condom social marketing [21] were used to assess the total of program-supported condoms. Other condoms available in AP through public and private social marketing and sales, and other public sector condoms were not tracked. Data on the number of condoms distributed to FSW every month and the estimated commercial sex acts were used to identify any gaps in condom need [Table 2]. The source where condoms were been last obtained by FSWs reported in the IBBA was compared with program monitoring data.

Other intensity measures examined were IBBA data on contacts by peer educators to assess outreach frequency and clinic MIS data on FSW visits to Avahan STI clinics [Table 2].

#### 2. Has there been an increase in condom use in high-risk groups?

Self-reported condom use behaviour from two rounds of IBBAs was used to assess changes in condom use with commercial and non-commercial partners of FSWs. Commercial partners of FSWs were: (a) occasional clients, who were unknown to FSWs; or (b) regular clients, who repeatedly visited the FSW and were thus known to her. Non-commercial partners included main regular partners or husbands or steady boyfriends of FSWs. Consistent condom use was defined as condom use every time and no reported unprotected sex acts was defined as condom use every time with both occasional and regular clients.

#### 3. Has there been reduction in STIs and new HIV infections?

Changes in STI prevalence were based on tests done on blood and urine samples collected from FSWs during IBBAs. Biological tests included syphilis serology using Rapid Plasma Reagin (RPR) and confirmatory *Treponema pallidum* Hemagglutination Assay (TPHA), and nucleic acid amplification (Gen-Probe APTIMA COMBO 2) of urine samples for chlamydia and gonorrhoea prevalence [18]. Any positive RPR test confirmed by TPHA was defined as reactive syphilis or lifetime syphilis; whereas RPR yielding titres ≥ 1:8 were defined as active or high-titre syphilis.

HIV-seropositivity was determined using a two-test algorithm using enzyme immunoassay (J. Mitra EIA Kit) [18]. As proxies for new HIV infections, HIV prevalence among newer FSWs, those who entered sex work in the last year, and among young FSWs (aged between 18 to 20 years) was examined.

#### 4. Is there an association between Avahan exposure and increase condom use and decline in STIs?

Exposure to Avahan interventions was based on self-reported exposure to three program core services during the last year: (a) ever or within the last year contacted by peer educators; (b) ever visited the program’s STI clinics; and (c) received condoms from peer educators. Data pooled from the two rounds of the IBBAs were used to examine associations between exposure to Avahan intervention (each service given above) and reported condom use outcomes and STIs, as defined earlier.

### Data Management and Statistical Methods

Double-data entry of district –level datasets was conducted using CSPro software (U.S. Census Bureau, Washington DC) for both rounds of IBBA. SPSS 14.0 statistical software (IBM, Somers NY) was used for data analysis. District-level data from each round were merged to generate state-level datasets for Rounds 1 and 2. Some analyses were performed using pooled data sets generated by aggregating the data from Rounds 1 and 2. Appropriate weights, in the district-level and state-level data-sets for both Rounds 1 and 2 were calculated and used for analysis [19]. Bi-variate and multivariate analyses were conducted using the complex samples module in SPSS 14. The Wald Chi-square test was used to assess significant changes in profile characteristics among FSWs between the two rounds of IBBAs. Multivariate logistic regression was used to generate crude odds ratios (ORs) to assess significant changes in: (a) exposure measures; (b) condom use outcomes with different partner types; and (c) prevalence of STIs and HIV between the two IBBA rounds; and additionally for studying associations between exposure to Avahan services and STIs (gonorrhoea, chlamydia or high-titre syphilis) and consistent condom use with commercial and non-commercial partners. Profile variables found to be significant in bivariate analysis between two surveys were adjusted in logistic regression models to generate adjusted odds ratios (AORs). Associations were considered significant for p-values below 0.05.

## Results

The findings are presented in the form of answers to the assessment questions shown in Table 2. A total of 6,496 FSWs (3,271 in Round 1 and 3,225 in Round 2) were sampled in the IBBAs [22,23]. The proportion of sampled FSWs by district is provided in Table 1 and the profile characteristics of FSWs for each round are provided in Table 3. Overall, 6% of FSWs in Round 2 reported that they had participated in Round 1 (Data not Shown).

**Table 3:** Socio-demographic and sex work characteristics of female sex workers in Andhra Pradesh in round 1 and 2 of IBBA

Characteristic	Categories	Round 1 %	Round 2 %	P value (Wald-Pearson test)
Number of respondent **(N)**		**3271**	**3225**	-

Response rates		74	58	-

Current age (years)	< 25	21.3	20.1	0.68
	25-29	24.9	28.1	
	30-34	21.6	20.1	
	35-39	19.8	19.8	
	40+	12.3	12.0	
	
	Mean	30.0	30.1	

Literacy	Illiterate	66.8	59.0	<0.01

Marital status	Currently Married	48.6	56.1	<0.01
	Ever married but currently not living with husband	43.6	34.6	
	Never married	7.8	9.3	

Additional Income	Yes	54.5	48.0	0.01

Residency	Local dweller	92.9	53.1	<0.001

Age at first sex (Years)	< 15	30.2	31.0	0.68
	15+	69.8	69.0	
	
	Mean	15.8	15.6	

Age started sex work (years)	<20	23.6	21.1	0.09
	20-24	31.5	32.7	
	25-29	26.7	24.4	
	30+	18.3	21.8	
	
	Mean	23.8	24.5	

Duration sex work (years)	0-1	12.5	16.1	0.01
	2-4	34.4	35.9	
	4-9	28.8	29.1	
	10+	24.4	18.7	
	
	Mean	6.2	5.6	

Usual place of solicitation	Home	31.9	17.0	<0.001
	Brothel/lodge/dabha	14.7	8.6	
	Public places	53.5	74.4	

Usual place of entertaining clients	Home	47.2	38.3	<0.001
	Brothel/lodge/dabha	37.1	20.3	
	Public places	15.6	41.5	

Commercial clients per week	0-4	21.2	11.7	<0.001
	5-9	38.5	37.3	
	10+	40.3	51.1	
	
	Mean	9.5	11.1	

Had Regular partner	Yes	76.4	71.6	<0.02

Source of obtaining condom	Peer educator/out reach worker/NGO	58.1	61.4	0.007
	Other source	41.9	38.6	

Frequency of contacts by peer educator in last one month	Visited once	22.0	12.1	0.045
	2-3 times	50.0	31.4	
	4 time or more	28.0	56.5	
	
	Mean	**2.9**	**5.3**	

### 1. Is coverage of Avahan adequate?

#### Scale of coverage

Avahan’s program in Andhra Pradesh was launched in 2004 in 17 districts and expanded to all 23 districts by 2007. By 2007, all districts were covered either by Avahan-supported services or Government of India-supported services. As the Avahan program expanded, the number of Avahan-supported NGOs increased from 25 in 2004 to 66 in 2009. The total estimated FSW population planned to be covered by Avahan as of March 2009 was 75,000 in all 23 districts combined.

**Figure 2:**
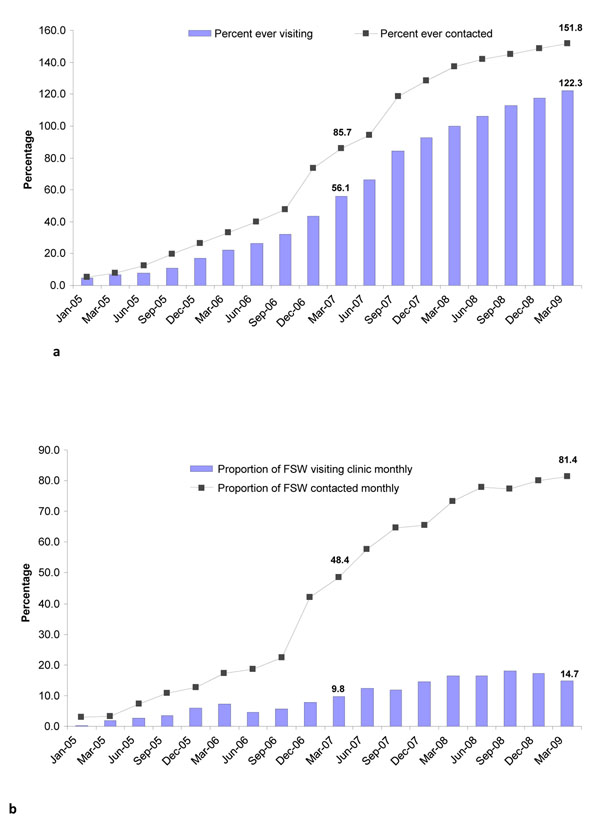
Figure 2a: Proportion of FSWs ever contacted and ever visited clinic in Andhra Pradesh (Avahan CMIS: 2005-2009). Figure 2b: Proportion of FSWs contacted and visited clinic in last month in Andhra Pradesh (Avahan CMIS: 2005-2009).

As per CMIS data based on 23 districts by March 2009, the Avahan program recorded a coverage of about 152% of estimated FSWs (114,000 FSWs) reached at least once through peer contacts and 122% (91,500 FSWs) through STI clinical services (Fig 2a). The percentage of the total denominator contacted monthly by peer educators increased consistently over the four years and reached 81% of estimated number of FSWs and while monthly visits to Avahan STI clinics by FSWs reached 15% of the denominator by March 2009 (Fig. 2b).

However, IBBA Round 2 data based on eight districts show that the proportion of FSWs who had ever been contacted by Avahan program peer educators was 44%, a significant decline from 49% in Round 1 (AOR: 0.73 p=0.04) and similar to the decline of FSWs who had ever visited Avahan STI clinics (from 49% in Round 1 to 40% in Round 2 [AOR: 0.48; p=0.001]). The proportion of FSWs who were contacted by peer educators in the last month in Round 1 was 42% (in five districts), whereas in Round 2 it was 32% (in all IBBA districts) (Data not shown).

#### Intensity of coverage

**Figure 3:**
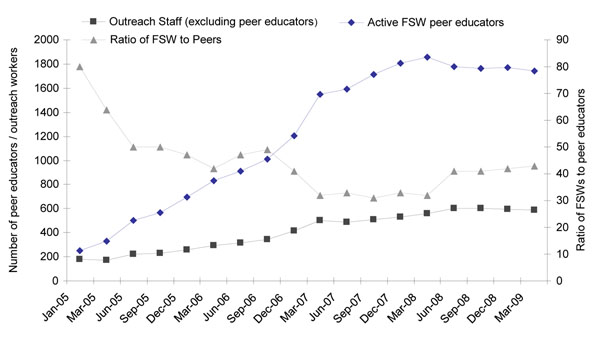
Number of active peers educators / outreach workers and ratio of FSWs to peers educators (Avahan CMIS 2005-2009).

The number of peer educators and outreach workers under the Avahan program increased and the target ratio of peer educator to FSWs (45 FSW/PE) was reached by December 2005 (Fig. 3). Free condom distribution by the program in Andhra Pradesh increased sharply from 1.3 million (2005) to 35 million (2009). Project-supported condom social marketing reached 2.2 million in 2005 and 3.6 million in 2008. The ratio of condoms per FSW increased from about 4 in 2005 to 31 in 2007 and to 45 in 2008, while the estimated volume of commercial sex acts per FSW per month remained at about 45 between 2006 and 2009. Thus, condom distribution by the program reached a level to cover all estimated commercial sex acts by 2008. In the IBBAs, FSWs reporting receipt of condoms from the Avahan program increased from 58% in Round 1 to 61% in Round 2 (p=0.01). (Data not shown).

**Figure 4:**
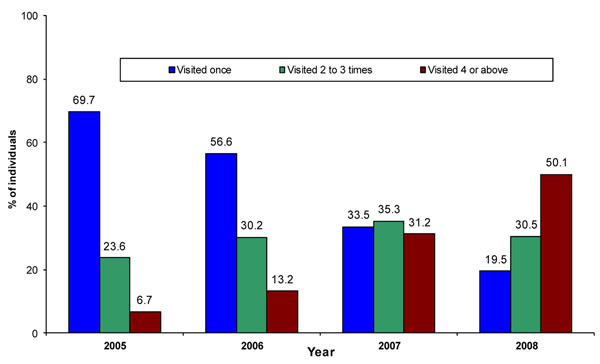
Frequency of visits to clinics in Andhra Pradesh (Individual STI clinic data 2005-2008).

The proportion of FSWs who reported being contacted more than once increased significantly (p<0.001) from 78% during IBBA Round 1 to 88% in Round 2 (Table 3). Clinic MIS data indicated more frequent uptake of clinical services, with the proportion of FSWs who made four or more visits to Avahan STI clinics increasing from 6.7% in 2005 to 50% in 2008 (Figure 4).

#### Quality of services

Assessments conducted in 20% of Avahan clinics (71 in 2005 to 110 in 2008) showed that the mean aggregated quality scores increased from 1.5 to 3.6 (p=0.13) out of maximum score of 5.0. The clinic service management score increased from 1.6 in 2005 to 4.2 in 2008 (p=0.13). Similarly, mean clinic utilization scores increased from 0.9 to 3.2 (p=0.51) and mean clinic operations scores increased from 2.0 to 3.8 (p=0.26) (Data not shown).

### 2. Has there been an increase in condom use among HRGs?

#### Change in condom use patterns over time

Univariate and multivariate analysis showed a consistent and significant increase in reported condom use with occasional (71% to 84%; AOR=2.2; p<0.001) and regular (64% to 83%; AOR=3.0; p<0.001) clients in Round 2 compared to Round 1 (Table 4). The proportion of FSWs who reported no unprotected sex acts with commercial clients increased from 59% in Round 1 to 82% in Round 2 (AOR=3.3; p<0.001). Condom use with regular partners remained low (9%) and unchanged during this period.

**Table 4:** Changes in intermediate condom use outcomes and HIV and STI prevalence among FSWs in Andhra Pradesh

	IBBA R1 N=3271	IBBA R2 N=3225	Crude OR (95% CI)	Adjusted OR (95% CI)	P value (Wald test)
**No reported unprotected sex with commercial clients in past month**	59.4	81.8	3.070** (2.49 - 3.78)	3.28 (2.57 - 4.18)	<0.001
**Condom use last sex act occasional clients**	91.3	97.8	4.18** (2.99 – 5.84)	4.10 (2.79 – 6.02)	<0.001
**"Every time" condom use with occasional clients**	70.8	83.7	2.19** (1.77 – 2.72)	2.20 (1.75 – 2.81)	<0.001
**Condom use last sex act regular client**	85.0	95.9	4.08** (2.90 – 5.76)	3.61 (2.40 – 5.44)	<0.001
**"Every time" condom use with regular clients**	63.6	83.4	2.88** (2.32 – 3.58)	2.98 (2.29 - 3.88)	<0.001
**Last time condom use with regular partner**	19.2	16.9	0.86 (0.55 – 1.32)	0.99 (0.68 – 1.43)	0.99
**"Every time" condom use with regular partners**	8.9	8.5	0.95 (0.59 – 1.53)	1.10 (0.66 – 1.85)	0.70
**HIV prevalence**	17.7	13.2	0.71* (0.52-0.96)	0.68 (0.51-0.90)	<0.01
**Syphilis infection**	10.8	6.1	0.54** (0.35 – 0.83)	0.39 (0.25-0.60)	<0.001
**High-titre syphilis**	3.2	3.1	0.98 (0.45-2.12)	0.45 (0.22-0.90)	0.03
**Chlamydia infection**	3.5	3.5	1.00 (0.69 – 1.45)	0.96 (0.61 – 1.49)	0.85
**Gonorrhoea infection**	2.2	2.8	1.28 (0.77 – 2.15)	1.22 (0.68 – 2.19)	0.49

**<0.01 *<0.05. Multivariate models were adjusted for the following variables: District, Literacy, Marital Status, additional sources of income, Locality status, Duration in sex work, Usual place of solicitation, Usual place of entertaining clients, No. of Clients per week and Proportion of FSWs with regular partners

### 3. Has there been a reduction in STIs and new HIV infections?

#### Change in STI prevalence and visits to clinics with STI symptoms over time

The prevalence of reactive syphilis serology among FSWs dropped significantly from 11% to 6 % (AOR 0.39; p<0.001) between Rounds 1 and 2 (Table 4). There was a marginal but statistically significant decline in high-titre syphilis between the two rounds (p=0.03) when adjusted for socio-demographic and sex work characteristics. Other STIs did not show any significant changes.

#### Change in HIV prevalence and new HIV infections

The overall prevalence of HIV infection among FSWs for all IBBA districts pooled, declined significantly from 17.7% to 13.2% (AOR 0.68; p<0.01), between Rounds 1 and 2 (Table 4). When analyzed district wise, significant decline was seen in only one district; while non-significant changes were seen in the remainder after controlling for confounding factors (Table 5). HIV prevalence among younger FSWs who were in the age group of 18-20 years, decreased significantly from 17.7% in Round 1 to 8.2% in Round 2 (p=0.008). However, HIV prevalence among FSWs reporting duration less than a year into sex work profession remained unchanged, 15.8% in Round 1 and 16.0% in Round 2.

**Table 5:** District wise HIV and syphilis prevalence among FSW in Andhra Pradesh

	IBBA RI N=3271	IBBA RII N=3225	Crude OR (95% CI)	Adjusted OR (95% CI)	P value (Wald test)
**HIV prevalence**					

**Chittoor**	8.1	10.5	1.34 (0.66-2.73)	1.20 (0.60-2.41)	0.61
**East Godavari**	26.3	23.3	0.85 (0.49-1.48)	0.81 (0.41-1.61)	0.55
**Guntur**	21.3	8.4	0.34** (0.19-0.62)	0.64 (0.27-1.52)	0.31
**Hyderabad**	14.3	9.6	0.64 (0.31-1.30)	0.55 (0.21-1.42)	0.22
**Karimnagar**	21.1	6.5	0.26** (0.13-0.51)	0.26 (0.09-0.71)	0.009
**Prakasam**	11.1	13.4	1.25 (0.57-2.72)	1.62 (0.52-5.09)	0.41
**Visakapatnam**	14.2	18.2	1.34 (0.47-3.81)	0.57 (0.29-1.12)	0.103
**Warangal**	10.8	15.0	1.45 (0.36-5.78)	0.90 (0.23-3.52)	0.884

**Syphilis prevalence**					

**Chittoor**	10.4	3.4	0.31** (0.13-0.71)	0.17 (0.06-0.49)	<0.001
**East Godavari**	15.0	18.0	1.24 (0.51-3.00)	0.47 (0.14-1.51)	0.20
**Guntur**	8.6	3.0	0.32* (0.14-0.72)	0.30 (0.11-0.81)	0.02
**Hyderabad**	17.4	6.7	0.34** (0.18-0.65)	0.31 (0.11-0.82)	0.02
**Karimnagar**	6.4	3.6	0.54 (0.20-1.45)	0.81 (0.29-2.26)	0.684
**Prakasam**	5.2	3.2	0.62 (0.19-1.96)	0.37 (0.07-1.94)	0.24
**Visakapatnam**	7.1	4.7	0.65 (0.28-1.49)	0.75 (0.27-2.08)	0.572
**Warangal**	10.2	1.5	0.13* (0.04-0.51)	0.04 (0.01-0.23)	<0.001

**<0.01 *<0.05. Multivariate models were adjusted for the following variables: District, Literacy, Marital Status, additional sources of income, Locality status, Duration in sex work, Usual place of solicitation, Usual place of entertaining clients, No. of Clients per week and Proportion of FSWs with regular partners.

**Table 6:** Association of Avahan program exposure variables with condom use outcomes and having any STI among FSWs in Andhra Pradesh

Indicators	Contacted by Avahan PE			Visited Avahan NGO clinic			Received condoms from Avahan PE		
	
	Yes (%)	OR (95% CI)	AOR (95% CI)	Yes (%)	OR (95% CI)	AOR (95% CI)	Yes (%)	OR (95% CI)	AOR (95% CI)
Condom use last sex act occasional clients	95.4	1.90** (1.44-2.49)	1.86** (1.39-2.49)	95.8	2.09** (1.59-2.75)	2.13** (1.61-2.83)	95.7	2.21** (1.68-2.90)	2.05** (1.55-2.73)

Consistent condom use with occasional clients	76.0	1.42** (1.17-1.71)	1.40** (1.14-1.72)	77.7	1.28** (1.07-1.54)	1.34** (1.10-1.64)	76.4	1.42** (1.18-1.71)	1.36** (1.11-1.66)

Condom use last sex act regular clients	89.9	1.55** (1.20-1.99)	1.37* (1.04-1.79)	90.9	1.73** (1.34-2.24)	1.52** (1.15-1.02)	90.1	1.64** (1.28-2.11)	1.45** (1.08-1.84)

Consistent condom use with regular clients	70.7	1.32** (1.08-1.61)	1.26* (1.02-1.55)	71.9	1.16 (0.95-1.41)	1.14 (0.92-1.42)	70.6	1.26* (1.04-1.52)	1.17 (0.96-1.44)

Condom use last sex act regular partner	18.6	1.63** (1.23-2.16)	1.66** (1.22-2.27)	20.6	1.57** (1.21-2.03)	1.73** (1.30-2.32)	18.7	1.61** (1.22-2.12)	1.64** (1.21-2.24)

Consistent condom use with regular partner	8.2	1.38 (0.95-2.01)	1.46 (0.98-2.17)	8.6	1.36 (0.95-1.95)	1.42 (0.96-2.10)	8.2	1.34 (0.92-1.95)	1.41 (0.95-2.10)

Any STI (NG or CT or high titer syphilis)	7.2	0.78 (0.55-1.12)	0.91 (0.66-1.25)	6.9	0.72 (0.51-1.02)	1.05 (0.77-1.42)	7.2	0.77 (0.54-1.10)	0.94 (0.68-1.30)

**<0.01 *<0.05. Multivariate models were adjusted for the following variables: District, Literacy, Marital Status, additional sources of income, Locality status, Duration in sex work, Usual place of solicitation, Usual place of entertaining clients, No. of Clients per week and Proportion of FSWs with regular partners.

### 4. Is Avahan exposure associated with increase in condom use and decline in STIs?

‘Last-time’ condom use with occasional and regular clients were associated with each of the three Avahan program elements between Rounds 1 and 2 (Table 6). Consistent condom use was more likely among FSWs who had been contacted by Avahan peer educators (AOR=1.4; p<0.001), had visited Avahan STI clinics (AOR=1.3; p<0.001), and who received condoms from Avahan peers (AOR=1.4; p<0.001). However, consistent condom use with regular clients, was more likely only among FSWs who were contacted by Avahan peer educators (AOR=1.3; p<0.05). With regular partners, last time condom use was associated with each of the three Avahan service exposures but not consistent condom use. (Table 6). Analysis of having any STI with exposure to each of the Avahan services revealed no significant association. (Table 6). One of the possible reasons for lack of significant association between exposure and having any STIs could be due to low levels of their prevalence and longer duration required to observe perceivable changes.

## Discussion

The paper presents for the first time an assessment of a large scale HIV prevention intervention by the Avahan program for FSWs in Andhra Pradesh. Based on the assessment of the program monitoring data, Avahan program in Andhra Pradesh achieved scale with intensity, coverage and quality. Condom use with clients reported by FSWs in the IBBAs increased while the prevalence of syphilis (but not other STIs) and HIV decreased, more so among younger FSWs.

As stated earlier, Avahan was not the sole player in all the districts, since the State AIDS Control Society implemented programs in some geographic areas of the same districts [12,24]. The program monitoring data by Avahan thus provided information on coverage only for the areas covered by it while IBBAs provided data for the district as a whole. The differences in the estimates of coverage achieved by Avahan, between program monitoring and IBBA sources is likely due the following reasons: high levels of FSW mobility and turnover in the IBBA districts, limitations of size estimates of FSWs, districts supported by Avahan transitioning over to NACO and the time period of IBBA Round 2 data collection.

The estimated number of FSWs to be covered was based on the district-level size estimation exercises conducted by each implementing partners in the district at different time points with a gap of 12 to 24 months, and by using different methodologies [16]. While there were no major events or catastrophes to cause any large migration of populations during the period of Avahan intervention, Andhra Pradesh is reported to have the third-largest level of inward migration [25] in addition 88% of FSWs have been estimated to move every two years with high degrees of cross-district mobility (averaging at least three moves every two years) [26].

The Avahan program transition plan began after March 2009, and two districts, namely Hyderabad, and Visakhapatnam, were transitioned to the State program [24] which could have affected the measurement of coverage during IBBA Round 2 in those districts. Additionally, problems encountered in the administration of questions regarding program exposure in some districts would have affected the measurement of coverage; thus leading to probable under-estimation of coverage achieved by the program. Other variables in the IBBAs regarding program coverage, as measured by proportion of FSWs who last obtained condoms from peer educators or another intervention (61% in Round 2), further strengthen this argument.

The number of free condoms distributed by the Avahan program increased during implementation and reached the estimated monthly requirement of condoms to cover the estimated number of commercial sex acts for each FSW. The efforts for scaling up the condom distribution and social marketing were also scaled up by the National Program during the same time [27] and reflected in an increase in the condoms availability above the required levels [24,28]. Evidence from assessment among clients of FSW in southern states also indicates increased condom availability in Andhra Pradesh through social marketing efforts [21].

Two rounds of independent IBBA data showed significantly improved condom use among FSWs with commercial clients. A large-scale study conducted in 13 districts of the state from 2003-2004 reported less than 50% consistent condom use by street-based FSWs with clients [29]. If this were considered a baseline (since it was measured prior to Avahan), the results from the two rounds of IBBAs indicate significant increases in condom use during commercial encounters. Other studies in Andhra Pradesh and nationally have shown increasing trends in reported condom use by FSWs and clients across the state [30-32].

Condom use with regular partners, however, continued to be low between the two rounds of IBBAs. Results from an earlier study in 2007 in 13 districts reported that 21% among program-unexposed and 38% among program-exposed FSWs used condoms with regular partners [30]. Similar low levels of consistent condom use with regular partners have been reported in other Avahan-supported states [33,34]. While the Avahan program focused on all types of partners of FSWs, the declining trend in condom use with regular partners indicates a major need for improving programmatic focus and adopting new strategies to make improvements in this area. Needless to say, as long as FSWs continue to have unprotected sex with regular partners, the risk of both infection and transmission of HIV and STIs will persist.

A significant reduction in syphilis prevalence was seen similar to other Avahan states [33-35]. Reduction in reactive syphilis serology and stable prevalence of chlamydia and gonorrhoea (estimated after controlling for confounding factors) also indicate a potential contribution of Avahan interventions in controlling STIs. Reduction in the prevalence of syphilis in Karnataka state was also associated with Avahan program [35,36]. Symptomatic and asymptomatic treatment, and monthly screening services provided at Avahan clinics have proven to be effective in reducing STI rates among sex workers elsewhere [17,37]. The program monitoring data indicate that FSWs were attending Avahan clinics for regular STI check-ups and treatment and the observed decline in clinic visits for STI symptoms is consistent with a declining trend of STIs and improved health seeking behaviour [38].

Between the two rounds of IBBA, HIV prevalence among FSWs in all districts combined, declined significantly. This decline was consistent with the state-level sentinel surveillance data among FSWs which has shown a decline in HIV prevalence from 20% in 2003 to 9.7%% in 2007 [12,24,3]. While an overall decline was observed, there were some districts where HIV prevalence did not decline. The lack of decline in HIV prevalence could be a result of a constant pool of HIV-infected FSWs in the state as reported above [16,26]. Such non-declining HIV prevalence could be an indication that the program coverage needs to be further improved, bearing in mind the rapid turnover and a need to focus on improving condom use beyond the current levels with commercial partners. The levels of condom use with regular partners in this context is also of concern and could be a contributing factor for non-declining HIV and need to be addressed more effectively. Other factors, such as increasing registration for anti-retroviral treatment (ART) among adults and women [24], and the misbelief that people on ART do not transmit HIV, could have contributed to this non-decline in prevalence. This needs to be confirmed through analysis of data on HRG and FSW registration for ART services, which presently is unavailable. Finally, the timeframe for repeated assessments is only three to five years, too short for major changes in HIV prevalence given the local epidemiology and increasing ART use. Having a reliable HIV incidence test would allow for a more accurate assessment of impact but would require larger sample sizes [39].

The data suggest a fall in HIV prevalence among younger FSWs. This is consistent with data from Cambodia that demonstrated a significant decline of HIV in younger FSWs and only after a few years of interventions [40]. The lack of decline in HIV among women new to sex work may indicates the program’s limited success in reaching new sex workers and is consistent with a non-declining trend of HIV prevalence in many districts. As suggested above, this may indicate that new sex workers may be HIV-positive upon entering sex work. It has been reported that women enter sex work due to limited economic options, death of or separation from partners [41], making them vulnerable, since they are more likely to lack negotiation skills and be at greater risk of becoming infected and subsequently transmit to their clients and regular partners.

This analysis of pooled IBBA data presented in this paper found a strong association between Avahan program exposure and use of condoms with commercial partners among FSWs exposed to each of three Avahan program services, similar to the results seen in other Avahan states [33-35]. This provides the evidence to support the argument that the programmatic strategies of Avahan resulted in the expected intermediate outcomes. Other peer-mediated interventions outside India as well as those implemented by Avahan in Karnataka state have shown similar association of exposure and condom use outcomes [35,36].

However, the analysis of STIs among exposed and unexposed FSWs in Avahan districts did not show any significant difference in prevalence between the Avahan-exposed and non-exposed FSWs, similar to results in Tamil Nadu [33] and Maharashtra [34], but unlike the results found in Karnataka [35]. One possible reason for this could be that the duration between assessments is too short to detect a decline. Studies from other parts of India such as the Sonagachi project reported declines in active syphilis from 4.8% to 1.2%, but only after nearly 10 years of intervention [42]. Levels of active STIs in IBBAs Round 1 and 2 were low (between 2% and 4%) and additional statistical power would possibly be needed to detect significant differences.

While the scope of the current paper was not to assess the impact of the Avahan among the general population, an initial analysis was done to examine the direction of the trends. Though Andhra Pradesh continues to be classified as a HIV high-prevalence Indian state, data from sentinel surveillance of pregnant women show a declining trend of HIV prevalence from 1.75% in 2005 to 1% in 2008 [25,41]. Similar declines have been seen among younger antenatal clinic attendees (15-19 years) from 2% in 2005 to 0.8 in 2008 [12,24]. Pregnant women attending prevention of parent-to-child transmission (PPTCT) clinics has been steadily increasing in Andhra Pradesh [43] and these data have become now accepted for examining HIV trends as a proxy for ANC attendees and the general population [44]. The PPTCT data show that HIV prevalence has declined from 1.7% in 2005 to 0.94% in 2008, suggesting a decline in new infections [1,3]. Further, these data are corroborated by findings from the National Family Health Survey (NFHS) which found HIV prevalence among general population was 0.8% and 1.2% respectively among women and men. [45]. These trends are suggestive of the impact of the interventions in the state, both Avahan and the Government programs, and may be further validated through advanced modelling analysis.

This study has several limitations. The first cross-sectional surveys were conducted nearly 14 months after initiation of Avahan program in the districts, and therefore cannot be considered a true baseline. The Avahan program in Andhra Pradesh was an implementation partner with the State program and therefore intended to increase the coverage of the FSWs in all the districts. The program MIS captured data on FSWs who were covered in Avahan covered areas only, while the sampling design of IBBA included the entire district. The Avahan implementation and evaluation design did not allow for any control groups, in consideration of the ethical issues of withholding STI services [9]. Given these limitations, a design appropriate for the current assessment which is feasible for a large scale-public health programs was used [13]. In the scenario where multiple interventions are aiming to reach vulnerable population, to rapidly scale up coverage, such designs using different sources of evidence, have been recommended [46-50] as alternative to randomized controlled trials.

The strengths of the current assessment are that it was based on the Avahan evaluation design and took a step-by-step approach and presented evidence along the program’s logic model, examined coverage, outputs, intermediate outcomes, followed by associations with the program exposure. This was done using program monitoring data and independent survey data for validation of trends, and provided evidence for program effectiveness based on the congruency of trends [13].

## Conclusions

Avahan implemented a program for FSWs in Andhra Pradesh in conjunction with the Government program and scaled up rapidly to achieve coverage in the context of a highly mobile target population; ensured adequate condom supply; delivered high-intensity peer and STI clinical services resulting in positive behavioural outcomes including increased condom use. Reduction or stabilization of STI prevalence and a reduction in HIV prevalence were also observed. The association of Avahan program exposure with positive sexual behaviours could have influenced changes in HIV and STI prevalence. The consistency of decline in prevalence of STIs and HIV with independent survey data since scale-up of the programs suggests program effectiveness and its likely impact among general population groups.

## Competing interests

The authors declare that they have no competing interests.

## Authors’s contributions

HR, VK, HR, PSPV and SK were responsible for data acquisition, performed the data analysis and prepared the manuscript draft. BGNV, PG, RA and RP (PI for studies) participated in the design of the study, contributed to the analysis, read and approved the final manuscript.
